# Conceptualizing and measuring migration policy change

**DOI:** 10.1186/s40878-015-0016-5

**Published:** 2015-12-01

**Authors:** Hein de Haas, Katharina Natter, Simona Vezzoli

**Affiliations:** Department of Sociology, University of Amsterdam, Nieuwe Achtergracht 166, 1018 WV Amsterdam, The Netherlands

**Keywords:** Database, Emigration, Immigration, Methodology, Migration policies, Restrictiveness

## Abstract

This paper outlines the methodology of DEMIG POLICY, a new database tracking around 6,000 migration policy changes in 45 countries between 1945 and 2014. The article conceptualizes the notion of migration policy change and presents the coding system used to operationalize policy content, changes in policy restrictiveness, as well as the magnitude of policy changes. The paper also discusses the potential of DEMIG POLICY to improve our understanding of the nature, evolution, and effectiveness of migration policies. Besides significantly extending the geographical and historical coverage of existing migration policy databases, DEMIG POLICY also tracks emigration policies in order to overcome the common ‘receiving country bias’ in migration research. By offering key insights into the main features of the largest migration policy database completed to date, this paper hopes to provide useful guidelines to improve future efforts to measure migration policies. Such improvement is crucial given the heated debates on migration policy effectiveness on one hand and the still limited empirical evidence on this issue on the other.

## Introduction

Over the past decade, there have been increasing efforts to compile migration policy databases and to measure the impact of migration policies (for an overview of recent projects, see Ellerman 2013). Although the interest in measuring migration policies has gained momentum, there is still a lack of fundamental methodological discussions about the inherent trade-off between the desire to achieve a large historical (longitudinal) and geographical (cross-sectional) coverage on the one hand, and the comprehensiveness of policies that can be examined on the other. Such trade-offs are common to most migration database endeavours, whether they track migration stocks, flows or policies. This paper seeks to provide transparency regarding the methodology adopted in the construction of DEMIG POLICY, the largest migration policy dataset publicly available to date (see http://www.imi.ox.ac.uk/data).

So far, most migration scholars have focussed on tracking *changes* in migration policies. In their pioneering work, Mayda and Patel (2004) collected migration policies for 14 OECD countries between 1980 and 2000, covering policies in the areas of labour migration, asylum, family reunification, and border control. This dataset was expanded by Ortega and Peri (2012) to include migration policies up to 2006 and for an additional country, Luxembourg. Covering a longer time period but a much smaller number of countries, the Immigration Policy database (ImPol) by Mezger and Gonzalez-Ferrer (2013) measures immigration policy changes of France, Italy, and Spain across five different migrant entry channels (irregular entry, short stay, family reunification, study, and work) since the 1960s. Other scholars have compiled databases focussing on a specific policy types. For instance, Hatton (2009) investigated asylum policy changes that occurred between 1981 and 1999 across the EU-15 area (except Luxembourg).

The above-mentioned databases that track changes in migration policies are very useful to understand the evolution of policies over time as well as within and across countries. Yet they are less powerful for *comparing* absolute levels of migration policy restrictiveness (or, conversely, openness). Recently, several database initiatives have aimed at measuring absolute levels of restrictiveness. The Immigration Policies in Comparison project (IMPIC) compares immigration policies in 33 OECD countries over the 1980–2010 period along a range of pre-defined indicators (Bjerre, Helbling, Römer, and Zobel 2014). Ruhs’ database (Ruhs 2011) covers 46 high and middle-income countries but is limited to policies regulating labour migration in one year 2009. The ongoing Immigration Policy and Law Analysis project (IMPALA, see http://www.impaladatabase.org/) seeks to achieve policy comparability across time and space by providing a measurement of migration policies over the 1960–2010 period for 25 immigration countries, again across pre-defined policy indicators (Gest et al. 2014). Notwithstanding the considerable comparative power of such absolute measures of restrictiveness, this method limits data collection and analysis to a pre-determined set of policy variables, which means that idiosyncratic, country-specific migration policies are missed out.

Given the advantages and disadvantages of both types of (change-tracking and comparative) databases, they can be highly complementary: For instance, comparative databases could be used to calibrate tracking databases by providing a baseline level of restrictiveness across a number of policy areas, while tracking databases can provide historical depth and country specific details to comparative datasets. However, the value of both tracking and comparative migration policy databases depends in the first place on the theoretical and empirical research questions guiding and motivating the data collection. There is no ‘one size fits all’ database – and unguided efforts to collect data ‘for the sake of collecting data’ are unlikely to succeed because they have no self-imposed constraints and format.

DEMIG POLICY has been constructed between 2010 and 2014 as part of the DEMIG project (*Determinants of International Migration: A Theoretical and Empirical Assessment of Policy, Origin, and Destination Effects*). It situates itself in the tradition of change-tracking databases, where the unit of analysis is a policy change occurring in a specific country and year. DEMIG POLICY tracks 6,505 migration policy changes in 45 countries,^1^ with over 90 percent of them recorded in the post-WWII period. The focus on policy change reflects the project’s ambition to generate new theoretical and empirical insights into the way states and policies shape migration processes in their interaction with other migration determinants in origin and destination countries (de Haas 2011). More specifically, DEMIG POLICY was meant to allow both an evaluation of the evolution of migration policies over time, and an empirical assessment of the effect of these policies on international migration. And policy effects are best evaluated at moments of policy change.

This paper aims to highlight vital methodological issues with regards to the construction of migration policy databases though sharing our experiences and insights gained in compiling DEMIG POLICY. By discussing the rationale for choices made, this paper seeks to answer two fundamental methodological questions: *Given limited human, financial, and data resources, how can reliable decisions on data collection and coding be taken? Given the inherent ambiguity and subjectivity of data collection and coding processes, how can a maximum level of consistency be achieved?* Indeed, efforts at constructing databases are an inherently selective and, to a certain extent, subjective process, involving numerous decisions on crucial issues such as the inclusion (and exclusion) of policy types, their categorisation, and the elaboration and implementation of a reliable coding system. As there is often significant room for ambiguity and no ‘objective’ way to overcome these challenges, maximising transparency about the decision-making on policy selection and policy coding is vital to increase the reliability (ensuring that codes reflect the real nature of the policy change) and consistency (ensuring that codes guidelines have been respected throughout) of the database and ensure the appropriate interpretation of subsequent analyses.

This paper offers insights into the main features of the largest migration policy database completed to date, but also hopes to provide useful guidelines for future efforts to improve the measurement and coding of migration policies. Such improvement is crucial given the heated debates on migration policy effectiveness on one hand and the still limited empirical evidence on this issue. Better migration policy data will enable more reliable, empirically informed assessments on the nature and evolution of migration policies, as well as their effectiveness in steering migration flows.

## DEMIG POLICY: conceptual background

Two concepts were central to the construction of DEMIG POLICY: *policy change* and *policy restrictiveness*. Indeed, the effectiveness of policies can best be assessed in moments of policy change and against a criterion, which, in our case, was restrictiveness: Does a change in migration policy restrictiveness affect migration in the intended way? The main inspiration for this approach were the databases established by Mayda and Patel (2004) and Hatton (2009) which tracked migration policy *changes* over time instead of trying to capture the characteristics of entire migration policy regimes at a given point of time (which is the aim of comparative databases). Instead of attempting to measure absolute levels of restrictiveness, they assessed whether a given policy change made the existing policy framework more or less restrictive. These databases, as well as the ImPol database (Mezger and Gonzalez-Ferrer 2013), also provided a transparent explanation of coding systems and decisions. DEMIG POLICY builds on these approaches and seeks to improve them by (i) providing an elaborate conceptualisation of migration policies; (ii) expanding the geographical (cross-sectional) and temporal coverage and including both immigration and emigration policies; (iii) disaggregating major policy changes into their individual policy measures; and (iv) specifying the migrant group targeted by each policy measure.

First, DEMIG POLICY is based on a broad definition of *migration policies* as “rules (i.e., laws, regulations, and measures) that national states define and [enact] with the objective of affecting the volume, origin, direction, and internal composition of […] migration flows” (Czaika and de Haas 2013: 489). This acknowledges that the aim of migration policies is not only to affect the *volume* of migration (the usual focus of public debates) but also their origin, direction and/or composition.

This definition informed our operationalization of migration policies in two ways: (i) DEMIG POLICY focused on ‘policies on paper’, that is, the laws, regulations, and measures enacted by states to regulate migration. Thus, the database disregarded policy discourses and the implementation of policies. This decision to focus on the legal aspects of migration policies has been adopted by most major policy databases (such as Mayda and Patel 2004; Ortega and Peri 2012; Bjerre et al. 2014; Gest et al. 2014), while other, more qualitative research projects have investigated the implementation of policies on paper (Infantino 2010; Eule 2014). To a limited extent, DEMIG POLICY included contextual information such as parliamentary debates, policy strategies, and action plans wherever it seemed relevant to understand the broader context in which decisions were taken. This information was retained as contextual evidence of policy-making processes, but was not coded as migration policy or included in analyses of policy effectiveness. (ii) Furthermore, only *national* policy measures were tracked despite the importance of regional and other sub-national policies in some countries, especially regarding integration. Supra-national (particularly European) regulations, such as the implementation of the Schengen and Dublin agreements, were recorded and coded within the respective national databases. We imposed these limitations mainly because DEMIG aims to cross migration policy data with flow data that is primarily available at the national level (Vezzoli et al. 2014).

Second, DEMIG POLICY expands the historical depth and geographical width of existing databases by covering migration policy changes in 45 countries from 1945 to 2014, albeit for some countries data reaches back to the late eighteenth century. To avoid a ‘receiving country bias’ and to enable measuring the effects of emigration policies on migration flows, DEMIG POLICY tracks both entry and exit policies for all countries included in the database. This also allowed us to move beyond any artificial and ambiguous separation between ‘sending’ and ‘receiving’ countries in the design of the database. Indeed, the data in DEMIG POLICY demonstrates that most countries are to some extent both and that many countries (for instance, in southern Europe and Latin America) have changed position over time, which is reflected not only in migration patterns but also in the evolution of their migration regimes.

The third innovation of DEMIG POLICY concerns migration policy disaggregation. Initial reviews of immigration and emigration policies (Czaika and De Haas 2013; de Haas and Vezzoli 2011) revealed that it is conceptually problematic to conceive of ‘a’ national migration policy, since migration changes are typically ‘mixed bags’ of often contradictory measures, liberalizing entry or stay rights for particular migrant groups while at the same time restricting access for other groups. This raises the fundamental conceptual and methodological problems of speaking of overall restrictiveness and makes it difficult to code change in restrictiveness of ‘entire’ policy changes (consisting of several measures targeting particularly migrant groups in quite different ways), which prior tracking databases have sometimes attempted (Mayda and Patel 2014; Ortega and Peri 2012). To overcome this problem, we decided to disaggregate policy changes into their different measures (some major migration policy changes consist of three, four or more measures) and code each measure separately instead of treating the entire policy change as one single data point. As an illustration, please refer to Table 1 further below (in section Data coding) to see how this disaggregation was operationalized in the database for Italy’s 2009 migration law. In this way, DEMIG POLICY acknowledges that most policies pursue a multiplicity of policy goals at the same time, which treat different migrant categories in very different ways, and are often incoherent *by design*.

**Table 1:** Excerpt from the DEMIG POLICY database

Country	Year	Policy change	Policy area	Policy tool	Target group	Target origin	Restrictiveness	Magnitude
Germany	1961	Labour agreement with Turkey - to recruit guest workers	Legal entry and stay	Recruitment programmes	Low-skilled workers	Specific nationalities (Turkey)	Less restrictive (−1)	Mid-level change (3)
South Korea	1975	Amendment to the Emigration Law of 1962 - to prevent high-ranking government officials, including judges and presidents of national corporations, from leaving	Exit	Exit visa/permit or exit ban	Skilled/high- skilled workers	Citizens	More restrictive (+1)	Mid-level change (3)
United States	1986	Immigration Reform and Control Act (ERCA or Simpson-Mazzoli Act) - introduced the General Legalization Program whereby unauthorized immigrants who were in continuous residence since January 1, 1982 were eligible for temporary legal status	Legal entry and stay	Regularisation	Irregular migrants	All foreign nationalities	Less restrictive (−1)	Major change (4)
India	2004	Citizenship (Amendment) Act 2003, came into force in 2004 - regulated citizenship for persons who are married to a citizen of India and are ordinarily resident in India for 7 years (5 years pre\iously)	Integration	Access to citizenship	Family members	All foreign nationalities	More restrictive (+1)	Fine-tuning change (1)
Italy	2009	Law 94 or Part II of the “Pacchetto Sicurezza” (“Security Package”) - made it possible to keep illegal immigrants up to 180 days (previously 60 days) in so- called Identification and Expulsion Centres	Border and land control	Detention	Irregular migrants	All foreign nationalities	More restrictive (+1)	Minor change (2)
Italy	2009	Law 94 or Part II of the “Pacchetto Sicurezza” (“Security Package”) - made conditions easier for foreigners graduating from an Italian university, who now have 12 months to find a job	Integration	Work visa/permit	International students	All foreign nationalities	Less restrictive (−1)	Mid-level change (3)

Fourth, given the variation of migration policy changes along migrant categories, DEMIG POLICY identifies and codes the migrant group targeted by each policy measure. The disaggregation of policy changes into different measures allowed to specify which migrant group was targeted by each measure, be it highly skilled workers, family members, refugees or irregular migrants. In this way, DEMIG POLICY offers the possibility to assess migration policy changes towards specific migrant groups over time and across countries. Through this code, DEMIG POLICY can identify policy changes that simultaneously open migration opportunities for some groups while reducing them for others, providing empirical evidence for the fact that modern migration policies are typically about selection. More generally, data in DEMIG POLICY can be aggregated across several indicators (for instance per year and country, per policy area, per migrant group etc.) which maximises the flexibility for quantitative and qualitative analyses on the evolution of migration policies.

## Data collection

It is an illusion to think that databases can be totally ‘objective’ or ‘neutral’ collections of information. Choices around selection of data, units of analysis, coding, the coverage of topics, countries and time-span are inevitable and should ideally be guided by theoretical concepts and research questions rather than by time and resource constraints. Yet there is an inevitable trade-off between coverage in terms of units of analysis (in our case countries) and years, and the level of detail and comprehensiveness in terms of the data collected for each unit of analysis in every year. This section lays out the rationale for such choices made in the data collection for DEMIG POLICY.

The compilation of DEMIG POLICY started with an extensive and systematic literature review of migration policies and of all reports of the OECD’s Continuous Reporting System on Migration (SOPEMI reports, since 2006 known as the OECD’s International Migration Outlook), published yearly between 1973 and 2013. The information collected through these reports was complemented by the systematic reading and evaluation of national migration profiles compiled by the Migration Policy Institute, the Migration Policy Centre, Focus Migration and the European Migration Network, as well as reports by international organisations, think tanks and NGOs. Whenever possible, information was cross-checked and complemented by academic articles on countries’ migration policy evolution and primary sources (legal texts, governmental documents) in the country’s original language.^2^

Besides entry policies, DEMIG POLICY also tracks border control, integration and exit policies. The decision to include integration policies was based on the consideration that post-entry rights may play a role (as deterring, attracting or retaining factor) in the migration decision of potential future migrants as well as migrants already in the country. For example, many governments create attractive conditions for family reunification and improve access to long-term residence or citizenship to attract high-skilled migrants, while restrictive access to social benefits is often used to deter future asylum and other ‘undesired’ migrants. The decision to include exit policies was partly motivated by our interest to test the effect of emigration policies on migration flows. These policies – which cover policy measures from expulsion to voluntary return programmes to ‘diaspora’ policies – are often ignored, but potentially important determinants of international migration.^3^ Together, this also enables to study immigration and emigration processes in relation to each other, that is, to investigate overall processes of migration and circulation and how these may be simultaneously influenced by entry, stay and exit policies.

The DEMIG project thus adopted a more inclusive definition of migration policy compared to other recent dataset construction efforts: For instance, the IMPIC dataset (Bjerre et al. 2014) focuses on admission policies, although it also includes deportation policies and some aspects of integration policies such as the right to work and access to welfare (while social and political rights are excluded). The IMPALA dataset (Gest et al. 2014) focuses on admission policies only, although it includes an indicator for naturalization policies. It does not make explicit whether border control measures and exit policies such as return programmes or deportation are also covered. Given the potential effects, either directly or in interaction with each other, of border control, entry, integration and exit regulations, DEMIG POLICY was set up to include these four policy fields. Figure 2 in the Appendix shows that 47 per cent of the policy changes recorded in DEMIG POLICY since 1945 deal with legal entry and stay issues, followed by 26 per cent addressing integration, 13 per cent dealing with border and land control and 12 per cent concerning exit regulations.

**Fig. 1:**
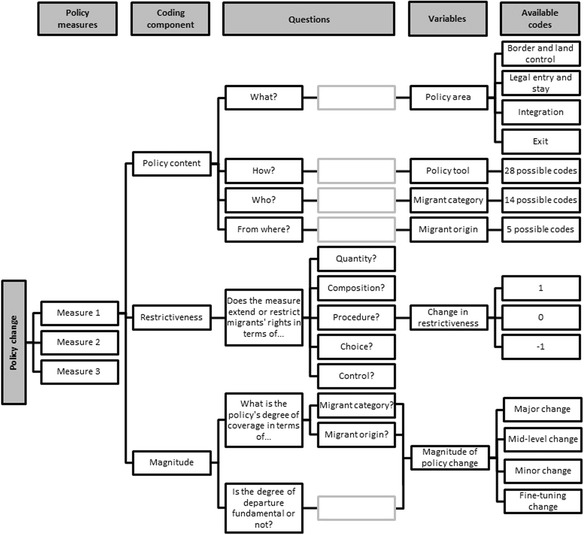
DEMIG POLICY coding scheme

Three types of policies were not tracked systematically: (i) ‘migration and development’ policies, which are often designed in cooperation with development agencies or with international and non-profit organisations, making this information scattered and inconsistently available; (ii) policies targeting unaccompanied minors and trafficking in human beings, as these often enjoy specific treatment despite them being a numerically small group; (iii) bi- and multilateral agreements related to migration, such as agreements on readmission, reintegration, trainee exchanges, or seasonal workers. The latter information is highly incomplete and achieving full coverage would not have been feasible with the resources available within the DEMIG project. Solely the traditional recruitment agreements from the pre-1973 period, for which detailed records exist in the literature, are tracked consistently.

The starting year of systematic data collection for DEMIG POLICY was set at 1945, since the end of World War II marked a turning point in migration patterns and migration regimes. However, pre-1945 information was included when easily available and relevant, and is particularly elaborated in the datasets of Argentina, Brazil, Canada, France, Mexico, New Zealand and the United States. As a result, a total of 6,505 policy changes are tracked in DEMIG POLICY, with 5,930 recorded in the 1945–2014 period. Pre-1945 data is incomplete, but it provides a basis for future historical extensions of the database.

Over the 1945–2014 period, there has been an increase in the number of policy changes recorded. On one hand, this may partly reveal the difficulty in identifying migration policy changes in the earlier historical periods. On the other hand, this surely also reflects a real proliferation of migration policies, including their frequent adjustments. This growth is particularly noticeable between 1985–1989 and 1990–1994, when the number of policy changes recorded more than doubled, and a further major increase in 2000–2004. The introduction of complex migration policy packages targeting multiple objectives and migrant groups are bound to result in the rapid growth in the number of policy changes. Figure 3 in the Appendix shows the distribution of policy changes recorded since 1945 per 5-year periods.

**Fig. 2:**
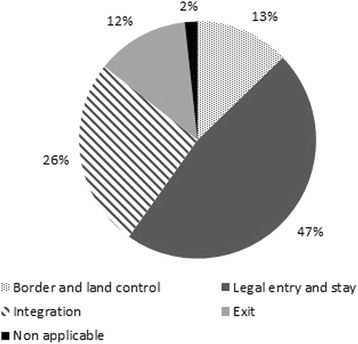
Distribution of policy changes by policy areas, 1945–2014

The choice of countries included in DEMIG POLICY was guided by three main considerations: (i) First, all major historical and current immigration countries were selected, such as Argentina, Australia, Brazil, Canada, France, Germany, New Zealand, South Africa, the United Kingdom and the United States, as well as traditional emigration countries such as Greece, Italy, Mexico, Morocco, Portugal, Turkey, or Spain. (ii) Second, availability of migration flow data in the DEMIG TOTAL and C2C databases also informed our choice, particularly when the data was of high quality, which led us to include countries such as Chile, Iceland and Luxembourg. (iii) Finally, institutional research experience of the International Migration Institute (IMI, University of Oxford), which hosted the DEMIG project, as well as individual researchers’ interests, led to the inclusion of particular regional migration hubs such as China, Russia, South Korea and Ukraine.

Figure 4 in the Appendix shows the distribution of policy changes recorded since 1945 across the 45 countries included in DEMIG POLICY. On average, 130 migration policy changes were recorded per country and for two thirds of the countries, between 90 and 170 changes were tracked. The largest number of policy changes were recorded for Canada and France, as well as other countries with long immigration histories such as the United States and Australia or that play a crucial role as regional migration hub, such as Germany and Spain. The lowest number of policy changes were recorded for Czechoslovakia, Yugoslavia and the German Democratic Republic, reflecting the fact that these countries ceased to exist before migration became a phenomenon of great importance. Surprisingly, some countries with high migration volumes, such as Luxembourg and Israel, have relatively few migration policy changes.

**Fig. 3:**
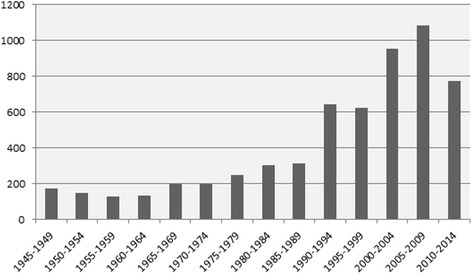
Number of policy changes recorded per 5-year period, 1945–2014

**Fig. 4:**
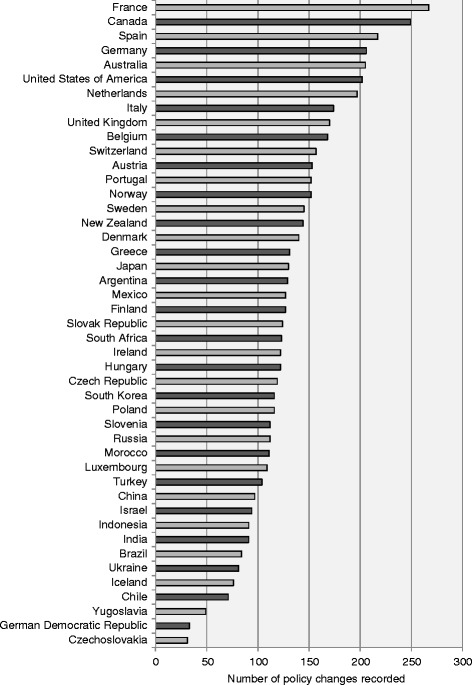
Number of policy changes recorded by country, 1945–2014

Given the geographical and historical spread of DEMIG POLICY, the diversity of data sources raised three main challenges: (i) First, collecting data on such a wide range of countries with various political and legal systems, as well as migration histories, presented considerable conceptual and linguistic challenges. (ii) Second, by relying on official reports, the policy changes tracked may be partly biased towards politically more salient policy issues in a specific country. (iii) Third, it was challenging to capture less recent policy changes because information on historic migration policies is generally less detailed. This is particularly true for the period before the 1970s (see Fig. 3 in the Appendix), as well as more generally for non-OECD countries (see Fig. 4 in the Appendix). Overall, this might have led to a bias towards historic policies which are retrospectively perceived as important because of their striking success or failure, or the numbers of migrants they affected.

However, by triangulating data sources, we have put significant efforts in preventing such biases as much as possible. In addition, every country dataset was reviewed by a national migration policy expert (see acknowledgements). Although the need for expert feedback varied across countries, the expert reviews proved to be a very valuable mechanism to include additional literature in the national language, improve the accuracy of the data, and increase the overall quality of DEMIG POLICY.

## Data coding

Coding exercises inescapably involve subjective decisions about definitions and categories, and personal biases may affect these decisions. Similarly, in elaborating the coding system and the definitions of respective codes for migration policy changes, the DEMIG team faced the dilemma of how to deal with migrant and policy categorizations that are commonly used in policy debates – such as ‘integration’ measures, ‘voluntary return’ programmes or measures towards ‘high-skilled workers’. While these categories may not always be sociologically meaningful, they may have become a lived reality for states and eventually also for migrants. The coding system adopted in DEMIG POLICY did not accept legal or conventional policy categories at face value, but attempted to use categories that can be conceptually justified.

Within DEMIG POLICY, each data entry represents one policy change enacted in a specific country and year. Each policy change has been coded by employing six main variables: Four variables capture the content of the policy measure (which policy area is covered, which policy tool used, and which migrant category and migrant origin are targeted); one variable assesses the change in restrictiveness of the policy (whether it makes the existing legal framework more or less restrictive); and one variable assesses the magnitude of the policy change (whether it represents a minor or major policy change).

Figure 1 presents the entire DEMIG POLICY coding scheme and Table 1 provides six examples of coded policy changes to exemplify it. The conceptual rationale and coding rules for each variable are outlined in the following sub-sections and will draw on these and other examples from the dataset.

### Coding policy content

The first crucial question is how to code the content or the substance of a policy measure. This coding decision inevitably involves a trade-off between the desire to grasp the full complexity of policy realities by operationalizing a multitude of nuanced codes, and the need for analytically useful, parsimonious categories by operationalizing a limited number of codes that capture the policy’s core elements. For instance, a policy measure introducing a labour market test to reduce the inflow of migrants in specific occupations could be given several codes corresponding to the targeted occupations, or a single code indicating whether this policy targets ‘low-skilled’ or ‘high-skilled’ workers. Depending on the aim of the database and the underlying research questions, one or the other option would be preferable.

The DEMIG POLICY coding system is based on the insight that most policy measures target particular categories of migrants rather than ‘all migrants’ and particular policy fields (e.g., border control or integration) rather than ‘migration’ in general. Thus, within DEMIG POLICY, the content of each policy measure is coded through four variables – two coding the issue addressed, two coding the group targeted:

1. The *policy area (what?)* variable identifies the broad field covered by the policy measure and consists of 4 codes.
2. The *policy tool (how?)* variable captures the instrument used to implement the policy measure and consists of 28 codes.
3. The *migrant category (who?)* variable specifies the migrant group targeted by the policy measure and comprises 14 codes.
4. The *geographical origin (from where?)* variable captures the origin of the targeted migrant category and comprises 5 codes.

Table 2 details the codes available for each variable. For each variable, the codes are mutually exclusive (e.g. a policy measure can only be coded either as regulating ‘border and land control’ or ‘legal entry and stay’). However, codes are non-hierarchical, therefore there are no fixed combinations of codes across the variables – for example ‘work visa/permit’ can be combined with either ‘legal entry and stay’ or ‘integration’ depending on the policy content.

**Table 2:** Variables to code policy content

Variables	Available codes
Policy area	Border and land control; legal entry and stay: integration; exit.
Policy tool	Surveillance technology/control powers; identification documents; detention; carrier liabilities; employer liabilities; other sanctions; travel visa/permit; work visa/permit; entry visa/stay permit; points-based system; quota target; regularization; entry ban; recruitment/assisted migration programmes; resettlement programmes; free mobility rights agreements; language_:_ housing, and cultural integration programmes; access to social benefits and socio-economic rights; access to justice and political rights; access to permanent residency; access to citizenship; reintegration return programmes; readmission agreements; expulsion; exit visa permit or exit ban; institutional capacities*; action plan, strategy, report*; and contextual elements*.
Migrant group	All migrants; all migrant workers; low-skilled workers; skilled high-skilled workers; family members; family members of high-skilled workers, investors, or students; family members of irregular migrants, or refugees, asylum seekers, and other vulnerable people; international students; investors, entrepreneurs, and business people; irregular migrants; refugees, asylum seekers, and other vulnerable people; members of the diaspora; and specific categories.
Migrant origin	All (both foreigners and citizens); all foreign nationalities; citizens; EU citizens; specific nationalities.

*These codes were included to improve the contextualization of policy measures and not assessed in terms of restrictiveness.

The examples in Table 1 above clarify this coding mechanism: For instance, Germany’s agreement with Turkey in 1961 to recruit guest-workers is coded as ‘regulating the *legal entry* through a *recruitment programme* of *low-skilled workers* from *specific nationalities (Italy)*’; while the first measure of Italy’s Law 94 of 2009 is coded as ‘regulating *border and land controls* through *detention* of *irregular migrants* from *all foreign nationalities’*.

While these two examples are clear-cut, other coding decisions were more ambiguous: Does a regularisation programme for irregular migrants, like the US 1986 Immigration Reform and Control Act (see Table 1), fall within ‘integration’ or ‘legal entry and stay’ regulations? Is the creation of reception centres for asylum seekers about ‘detention’, ‘language, housing, or cultural integration programmes’ or about ‘institutional capacities’? Is the introduction of a labour market test for all migrant workers relating to ‘employer liabilities’ or ‘work visa/permits’? And should a policy targeting care workers, without further definition of the workers’ qualifications, be coded as ‘skilled/high-skilled workers’ or ‘low-skilled workers’? In order to minimize arbitrariness, the process of coding policy content was guided by the five following rules:

1. *First, the code should reflect the explicit text of the policy measure, not our subjective interpretation of its underlying, alleged implicit or ‘hidden’ political intentions.* For instance, the creation of reception centres for asylum seekers was coded ‘institutional capacities’ in DEMIG POLICY, as this corresponds most accurately to the policy description available – using another code would require to subjectively assess whether the policy intends to foster integration or, on the contrary, to increase surveillance of asylum seekers.
2. *Second, we elaborated a detailed definition for each code, which was strictly applied in the coding decisions to ensure a maximum of consistency.* 4 For example, the introduction of a labour market test was coded as ‘work visa/permit’ because our definition of this code included “measures that establish, change, or abolish the procedures or eligibility criteria to obtain a work visa or permit before or after arrival”. In the same vein, care workers were coded as ‘low-skilled workers’ because the definition of this code specifically includes workers “who will work in occupations that do not require more than secondary education”. Despite the fact that care workers are often highly qualified individuals, the performed tasks do often not require higher education and are thus effectively in low-skill jobs.
3. *Third, the codes reflect the state perspective and not the migrant perspective, whenever this is relevant.* For instance, we decided to code the regularisation of irregular migrants as ‘legal entry’ and not ’integration’, as from the state perspective this measure is about giving people legal access to the country, even if they might already have lived in the country for several years.
4. *Fourth, a measure which seeks to expand or restrict the rights of a specific group, but which de facto affects a broad category of individuals, was coded with the more generic code.* This was for example the case with several border control measures that authorized police and other state organs to conduct random passport checks on the streets in order to detect irregular migrants. Although the aim of these types of policies to detect irregular migrants, it affects a broader part of society and was hence coded as targeting ‘all’ (including travellers, permanent residents, migrants and citizens), de facto all those who are assumed to be potential irregular migrants.
5. *Finally, in cases where two different codes would have been justified, coding decisions were discussed among members of the DEMIG POLICY team and recorded in the detailed coding protocol* 5 *to assure transparency and reproducibility* of the database*.*

Even with these coding rules, the biggest challenge was to ensure the consistent application of the codes throughout the database, particularly because the same code can have different meanings across languages and in different national contexts. For instance, although all points-based systems attribute points to specific migrant characteristics and all recruitment programmes imply state involvement, in practice the codes can reflect very different selection systems. No matter how detailed and objective the coding system and the definitions of the respective codes are, the actual coding exercise always involves interpretation, which creates potential biases. The elaborate DEMIG POLICY coding system, which was gradually developed through extensive internal discussions and several test coding rounds, served to minimize such biases and to maximize consistency.

### Coding changes in restrictiveness

The DEMIG POLICY database was constructed with the primary objective to test the effectiveness of migration policies in regulating migration. To assess policy effectiveness, it is first necessary to determine the aims of each policy measure. DEMIG POLICY used the *change in restrictiveness* introduced by the policy measure as a yardstick variable to assess the policy’s aim. For instance, the second measure of Italy’s law 94 of 2009, which allowed foreign graduates of Italian universities to stay for one year in order to find employment, introduced a change towards less restrictiveness, while South Korea’s 1975 law to prevent high-ranking government officials from leaving introduced a change towards more restrictiveness in the country’s migration regime (see Table 1). Because the code captures the *change* in restrictiveness introduced by the new policy measure *compared to the previous situation*, it is an ordinal variable assessing the relative change in restrictiveness in a specific policy field. It is not an assessment of the absolute level of restrictiveness of a specific policy within a country. One of the drawbacks of this system, common to other change-tracking migration policy databases, therefore is that this variable is not easily comparable across countries. On the other hand, this system offers considerable scope to compare policy trends over time across various countries, differentiating between different types of policies and targeted migrant groups (cf. de Haas et al. 2014).

The variable *change in restrictiveness* can take three values [−1, 0, +1] and the basis for deciding whether a policy introduced a more or less restrictive change was whether it extended or restricted the rights attributed to the targeted migrant group. Measures intending to restrict the rights of a migrant group were coded +1 (more restrictive than before), while measures intending to extend the rights of a migrant group were coded −1 (less restrictive than before). The code 0 (no change in restrictiveness) was used for the following two situations: (i) when a completely new selection system was introduced whose restrictiveness cannot be compared to the previous legal framework – such as the introduction of a points-based system into a labour migration regime that was previously demand-driven; (ii) for measures whose impact on rights cannot be assessed because changes affect non-coded dimensions (particularly age and gender groups).^6^

However, the disaggregation of policy changes into its different measures within DEMIG POLICY, as well as the comprehensive coding system, allowed for the large majority (78 per cent) of policy measures to be coded as either −1 or +1. 47 per cent of policy changes recorded in DEMIG POLICY since 1945 have introduced a change towards less restrictiveness, while 31 per cent have introduced a change towards more restrictiveness. While this indicates that the overall direction of policy change has been a liberalizing trend (see also De Haas et al. 2014), Fig. 5 in the Appendix shows that, in recent years, the proportions of restrictive and liberalizing policies have become more balance.

**Fig. 5:**
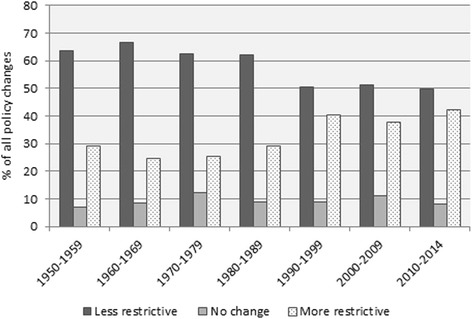
Distribution of policy changes by restrictiveness, 1945–2014

To minimize the potentially arbitrary character of coding, the DEMIG team elaborated the following five criteria to assess changes in policy restrictiveness:

1. *Quantity*: Does the measure restrict (+1) or widen (−1) the pool of (potential) migrants gaining entry, stay or exit rights?
2. *Composition*: Does the measure raise/specify (+1) or lower/make more generic (−1) the eligibility criteria for entry, stay or exit of a particular migrant group?
3. *Procedure*: Does the measure make specific procedures for entry, stay, or exit more (+1) or less (−1) complicated for the targeted group?
4. *Choice*: Does the measure restrict (+1) or widen (−1) the choices available to migrants?
5. *Control*: Does the measure increase (+1) or relax (−1) the level of control on migrants at the border or within the territory?

For example, when looking at Italy’s law 94 of 2009 (Table 1), the first measure that increased the duration of detention of irregular migrants introduced a restrictive change on the basis of the ‘control’ criterion, while the second measure granting a one-year job-search visa for foreign graduates of Italian universities introduced a change towards less restrictiveness on the basis of the ‘choice’ criterion. However, these five criteria are not mutually exclusive and in some cases the change in restrictiveness of a policy might be evaluated according to two or more criteria, such as in the example of India’s 2003 Citizenship Act, which increased the residency criteria for naturalization of foreign spouses to seven years and herewith introduced a restrictive change on both the ‘quantity’ and the ‘composition’ criterion.

### Coding the magnitude of change

Because not every policy change is equally important, we also included a variable to capture the *magnitude of change* introduced by the policy measure. This variable assesses whether a particular policy change constitutes a ‘fine-tuning change’, ‘minor change’, ‘mid-level change’ or ‘major change’, respectively coded 1, 2, 3 or 4. A two-step decision making process was used to determine the magnitude of change introduced by a policy measure. First, by assessing the *degree of coverage* of the policy change, i.e. whether the policy targets an entire migrant group or only part of it. Second, by assessing the *degree of departure* (fundamental or not) from the previous policy framework. Coding the magnitude of change was particularly challenging given the high reliance on subjective assessments on what a ‘fundamental’ change and ‘full coverage’ meant. This section outlines the detailed coding rules we elaborated to minimize the arbitrariness and subjectivity.

First, the *degree of coverage* evaluates whether the policy measure targets an entire migrant category or only part of a migrant category. As two codes determine the target group – geographical origin and migrant category (see section Coding policy content) – both were taken into account: (i) Policies targeting specific nationalities were automatically treated as affecting only part of a migrant category, while policies targeting all foreign nationalities, all citizens, or both together were treated as affecting an entire category. In the European context, policies targeting EU citizens only were also treated as an entire category due to the relevance of this category in policy making and the near-equal status of EU citizen with national citizens. (ii) Policies regarding generic migrant groups such as ‘all migrant worker’, ‘all family members’ or ‘all irregular migrants’ were considered as targeting an entire migrant category, while policies on sub-groups such as ‘low-skilled workers’, ‘rejected asylum seekers’ or ‘investors’ were considered targeting only part of a migrant category. By combining the geographical origin and migrant category targeted by the policy measure, we can determine whether a policy measure affects an entire migrant group or only part of it. Table 3 outlines the procedure of coding decisions.

**Table 3:** Assessing the degree of coverage of a policy measure

		Migrant origin	
		All foreign nationalities, Citizens, EU citizens	Specific nationalities
Migrant category	All migrant workers, all family members, all irregular migrants, All asylum seekers	Case 1: Targeted group covers entire migrant category	Case 3: Targeted group covers part of a migrant category
	Skilled/high-skilled workers, spouses, rejected asylum seekers, irregular workers	Case 2: Targeted group covers part of a migrant category	Case 4: Targeted group covers part of a migrant category

Some of the examples mentioned in Table 1 help to clarify how the degree of coverage criteria is operationalized: For instance, the 1961 recruitment agreement between Germany and Turkey is treated, from the German perspective, as targeting only part of a migrant group, as it is covering only low-skilled workers from one specific country (case 4 in Table 3). From the Turkish perspective, it is also treated as targeting part of a migrant group, but for a different reason, as although it is open to all citizens, it is in fact only covering low-skilled workers and not all workers (case 2). The regularization programme introduced by the United States in 1986 targeted an entire migrant group, that is, all irregular migrants from all nationalities (case 1). If the regularization would be open only to Mexican irregular migrants, however, it would be coded as targeting only part of the irregular migrant group (case 3).

Second, the *degree of departure* captures whether the policy measure introduces a fundamental change in the existing policy framework or not. In our definition, a fundamental change reflects the introduction or removal of a policy instrument, whereas a non-fundamental change reflects the modification of the characteristics of existing policy instruments or a continuation in the existing policy. For instance, the creation of a new entry permit, the granting of appeal rights, or the abolishing of borders are regarded as fundamental changes, whereas changes in age requirements for family reunification, in the administrative procedures for refugee status determination, or the broadening of eligible categories to an existing permit (such as increasing the number of professions exempted from a labour market test) are considered as non-fundamental changes. Thus, the 1961 German recruitment agreement or the South Korean exit ban for high-ranking government officials in 1975 are considered fundamental changes, as they open new entry channel for migrants (or abolish them), while the Indian law that increased residency requirements for naturalization of family members in 2004 or Italy’s decision to prolong the detention of irregular migrants are considered non-fundamental changes.

Once the degree of coverage and degree of departure of a policy measure are determined, it is possible to conclude on the magnitude of change introduced by this specific measure in the existing legal framework. Table 4 shows how the two-step decision-making process outlined above is combined to decide whether the policy change represents a ‘fine-tuning’ measure, a ‘minor’, ‘mid-level’ or ‘major’ change.

**Table 4:** Assessing the magnitude of change of a policy measure

		Degree of departure	
		Non-fundamental change or continuation of existing policy	Fundamental change of existing policy
Degree of coverage	Part of respective migrant category affected	1 - Fine-tuning	3 - Mid-level change
	Entirety of respective migrant category affected	2 - Minor change	4 - Major change

Referring to the examples of Table 1, the Indian Citizenship (Amendment) Act 2003 is coded as a ‘*fine-tuning measure’,* as it targets only part of a migrant group (spouses of Indian citizens from all nationalities, corresponding to case 2 in Table 3) and introduces a non-fundamental change in the existing legal framework (i.e. raising the length of residency required for naturalization). The first measure of Italy’s 2009 law 94 is coded as a ‘*minor change’*, as it targets an entire migrant group (all irregular migrants from all nationalities, corresponding to case 1 in Table 3), but introduces a non-fundamental change (i.e. prolonging the possible detention period). Germany’s 1961 recruitment agreement was coded as a ***‘*** *mid-level change* ***’***, as it targets part of a migrant group (only low-skilled workers from Turkey, corresponding to case 4 in Table 3), but introduces a fundamental change in the existing legal framework (i.e. the creation of a new entry channel). Finally, the 1986 regularization programme of the US Immigration Reform and Control Act was coded as a ***‘*** *major change* ***’***, as it targets an entire migrant group (all irregular migrants from all nationalities, corresponding to case 1 in Table 3) and introduces a fundamental change (i.e. the creation of a new entry channel).

However, coding the magnitude of a policy change can be ambiguous, especially when knowledge on the actual impact of the measure was available in retrospect. One of the working hypotheses within DEMIG was that relatively minor measures can have major effects whereas some major policy changes might have small or no effects. Using contextual and retrospectively available information to code the magnitude of a policy change would have introduced a major source of ‘endogeneity’ in our database. We therefore decided to code policies exclusively based on the policy content as recorded in the dataset and to discard and ignore any additional knowledge we might have had on the apparent impact of a policy measure or on the specific country context. The 1961 German-Turkish recruitment agreement provides a good example: In DEMIG POLICY, this agreement is coded as a ‘mid-level’ change because it targets low-skilled migrants from one nationality only. One would be inclined to code this measure as a ‘major’ change given its apparent huge impact on Turkish migration flows to Germany. The lack of information on policy effects and the concomitant inability to make such assessments for all policies, e.g. the 1996 agreement between Greece and Albania organizing the recruitment of seasonal workers, would have generated inconsistencies. Yet the most fundamental reason for exclusively coding on the basis of policy content is that the database tracks the nature of policies and that assessing the *impact* of these policies is the very goal of the subsequent empirical analyses using migration flow data. To maximize coding consistency across the database, the coding task was carried out by one person and reviewed by two team members. Difficult coding decisions were discussed collectively and decisions were tracked in the coding protocol.

## Conclusion

This paper outlined the rationale, considerations and principles that guided the collection and coding of migration policy changes in the new DEMIG POLICY database. Building upon prior efforts by other researchers, DEMIG POLICY introduced four innovations: (i) the inclusion of emigration policies to overcome the ‘receiving-country-bias’; (ii) the disaggregation of policy changes into their individual policy measures and the specification of targeted migrant groups, based on the fundamental insight that migration policies are typically ‘mixed bags’ of measures targeting various migrant groups in different ways; (iii) an elaborate conceptualisation of changes in restrictiveness, based on whether a policy change increases or decreases migrants’ access to rights in relation to the previous policy; and (iv) the operationalization of a variable that captures whether a policy changes represents a major or a minor change.

Compared to prior change-tracking databases, DEMIG POLICY expands the coverage in terms of countries, years and types of policies, as well as advances conceptual sophistication in terms of policy coding. Based on the methodology outlined in this paper, expanding the dataset back into history and including more non-Western countries are among the promising avenues for future improvement. As this paper argued, there is also considerable scope to combine change-tracking databases like DEMIG POLICY with comparative databases such as IMPALA and IMPIC that aim to measure absolute levels of restrictiveness or openness of migration policies through the development of indices. For instance, there seems to be a potential to use comparative databases to calibrate change-tracking databases by providing a baseline level of restrictiveness across a number of policy areas for specific years, which would increase the ability to use change-tracking databases for comparative purposes. In this sense, both tracking and comparative policy database have their distinct value, and should ideally be combined in future research.

Some of the lessons learnt through the construction of DEMIG POLICY seem more generally relevant for the construction of policy databases. First of all, our experience highlighted that we have to abandon positivist illusions of a coding system that ‘objectively’ tracks ‘policy facts’. ‘Policy facts’ do not objectively exist, but are always social constructions. Also, categorizations reflect a certain perception of the world and thus there is a danger in accepting existing legal and policy categories at face value without critically examining them. To reduce subjective biases, such as the tendency to select politically relevant policies and code them accordingly, it is important to carefully base decisions on theoretical considerations.

Furthermore, migration policy coding systems should be guided by underlying research questions and hypotheses: In their essence, coding systems are about synthetizing and condensing the types of information needed for analysis in order to answer specific research questions. By definition, simplification leads to a loss of nuance and complexity and should take place in a transparent manner through clearly defined and operationalized central concepts (such as migration policy, policy change and restrictiveness), conceptually informed categories and coding processes. Finally, policy data compilation as well as analysis will be strongest if embedded in a thorough understanding of the context in which those policies have emerged. This can avoid misinterpretation of quantitative results or ‘blind’, unguided efforts at ‘collecting more data’ without having in mind what purpose such data collection should serve in the first place.

As the largest migration policy database publicly available to date (see http://www.imi.ox.ac.uk/data), DEMIG POLICY is a valuable tool for analyses on the nature, drivers, and impacts of migration policy changes and is suitable for quantitative and qualitative analyses on the way in which migration policies affect and are affected by changes in the volume, composition, timing and direction of migration flows. The rich migration policy data included in DEMIG POLICY can also serve as a resource in itself for analyses about the evolution of migration policies in general or for particular types of policies or target groups.

For instance, can we really say that migration policies have become more restrictive over the past decades, as is often assumed in public and academic debates? Or do we rather see a broad trend towards increasing inclusiveness in which, as hypothesized by several scholars, human rights considerations and international law have compelled liberal-democratic states to expand possibilities for family and humanitarian migration (Bonjour 2011; Freeman 1995), in spite of restrictive migration discourses used by politicians suggesting the contrary? Or do we observe a more complex picture, with policies towards particular groups (such as asylum seekers or low-skilled labour migrants) becoming more restrictive, and less restrictive towards other groups (such as the high-skilled or students)? Or has the evolution of migration policies followed more erratic patterns with levels of restrictiveness oscillating in accordance with, for instance, economic cycles and the political colours of governments? And does the database allow uncovering policy ‘fashions’ in particular periods, with countries adopting similar measures in a policy diffusion process?

Finally, DEMIG POLICY has attempted to partly overcome the receiving country bias by not categorizing countries as either origin or destination countries – acknowledging that all countries are both –, by consistently tracking exit policies and by including several non-OECD countries in the database. There is ample potential for future extensions of the database in terms of geographical and historical coverage, so that in the future we may be able to better understand the long term evolution of migration regimes, and how changes in migration policies are related to broader transformation processes of such as colonization, state formation and economic restructuring. This would allow building upon the initial idea of DEMIG POLICY to go beyond rather narrow analyses of ‘migration policy effects’ and to develop a broader view on the historical role of states and policies in migration processes.

## Appendix 1

Distribution of policy changes by policy areas, 1945–2014

## Appendix 2

Number of policy changes recorded per 5-year period, 1945–2014

## Appendix 3

Number of policy changes recorded by country, 1945–2014.

## Appendix 4

Distribution of policy changes by restrictiveness, 1945–2014.

## Footnotes

1 Argentina, Australia, Austria, Belgium, Brazil, Canada, Chile, China, Czech Republic, Czechoslovakia, Denmark, Finland, France, Germany, German Democratic Republic, Greece, Hungary, Iceland, India, Indonesia, Ireland, Israel, Italy, Japan, Korea, Luxembourg, Mexico, Morocco, Netherlands, New Zealand, Norway, Poland, Portugal, Russia, Slovakia, Slovenia, South Africa, Spain, Sweden, Switzerland, Turkey, Ukraine, United Kingdom, United States of America, and Yugoslavia.

2 The database is accompanied by a comprehensive bibliography containing all sources used for each country. The complete sourcebook is available online at http://www.imi.ox.ac.uk/data. Note that the DEMIG research team reads English, French, German, Spanish, Portuguese, Italian, Dutch, Indonesian and Arabic.

3 We are aware of the fact that exit policies targeting citizens and foreigners are very different in nature and follow distinct objectives. Although both types of policy changes are coded as ‘exit’ policies, they can be identified and analysed separately by filtering through the target group.

4 The codebook with the detailed definitions of all codes is available online at http://www.imi.ox.ac.uk/data.

5 The coding protocol is available upon request from the authors.

6 We decided not to include variables to capture gender and age groups mainly because the large majority of migration policies does not use age or gender in their selection criteria. Given that both gender and age variables would have portrayed very little variation, they would have remained meaningless in subsequent analyses.
